# Self-tests for influenza: an empirical ethics investigation

**DOI:** 10.1186/s12910-017-0192-y

**Published:** 2017-05-05

**Authors:** Benedict Rumbold, Clare Wenham, James Wilson

**Affiliations:** 1 Department of Philosophy, University College London, Gower Street, London, WC1E 6BT UK; 2 LSE Health and Social Care, Cowdray House, London School of Economics and Political Science, Houghton Street, London, WC2A 2AE UK

**Keywords:** Self-tests, Ethics, Empirical ethics, Influenza, Point-of-care tests, Diagnostics

## Abstract

**Background:**

In this article we aim to assess the ethical desirability of self-test diagnostic kits for influenza, focusing in particular on the potential benefits and challenges posed by a new, mobile phone-based tool currently being developed by i-sense, an interdisciplinary research collaboration based (primarily) at University College London and funded by the Engineering and Physical Sciences Research Council.

**Methods:**

Our study adopts an empirical ethics approach, supplementing an initial review into the ethical considerations posed by such technologies with qualitative data from three focus groups.

**Results:**

Overall, we map a range of possible considerations both for and against the use of such technologies, synthesizing evidence from a range of secondary literature, as well as identifying several new considerations previously overlooked.

**Conclusions:**

We argue that no single consideration marks these technologies as either entirely permissible or impermissible but rather tools which have the potential to incur certain costs and benefits, and that context is important in determining these. In the latter stages of the article, we explain how developers of such technologies might seek to mitigate such costs and reflect on the possible limitations of the empirical ethics method brought out during the study.

**Trial registration:**

Not applicable.

**Electronic supplementary material:**

The online version of this article (doi:10.1186/s12910-017-0192-y) contains supplementary material, which is available to authorized users.

## Background

The past few decades have witnessed a series of significant advances in health diagnostic technologies, allowing for testing to be carried out rapidly at the point of care and by patients themselves (self-testing). Point-of-care tests now exist for many diseases and medical specialties, including: cholesterol [1]; glucose [2]; faecal occult blood and liver function tests [3]; HIV antigen and HIV antibody [4]; chlamydia [5]; cryptococcus [6]; malaria [7]; hepatitis C [8]; tuberculosis [9, 10]; blood alcohol level and drugs abuse. Although not available for as many health conditions as point-of-care diagnostics, there are also a growing number self-testing kits, including those for glucose, cholesterol, blood pressure, HIV-infection, fertility and pregnancy [11].

The potential benefits of such advances are evident. Point-of-care tests allow clinicians to receive test results much faster than traditional laboratory-based methods, improving patient care and clinical outcomes [12]. Laboratory-independence also means such tests can often be conducted in diverse settings, most notably those with weak health service or laboratory infrastructure [13]. Self-tests might also enable individuals to manage their health proactively, allowing individuals to make lifestyle changes or seek medical interventions sooner that could improve prognosis and reduce harm or suffering, as well as decrease infectious disease transmission [14].

Yet such technologies also raise ethical concerns. As with any diagnostic, kits may generate false positives or false negatives – potentially leading users to make decisions based on inaccurate information [15, 16]. With self-tests, risks are compounded by the increased likelihood of individuals failing to conduct the test properly, misunderstanding test results or misunderstanding the reported accuracy of those results [17, 18]. When tests are conducted at home, individuals are also less likely to have sufficient support structures in place, either pre- or post-diagnosis [14, 19, 20].

In this article we consider the ethical challenges posed a new set of diagnostics: namely, self-test diagnostic kits for influenza. We centre our analysis on one mobile phone-based diagnostic tool in particular, namely a new kit being developed by i-sense, an interdisciplinary research collaboration based (primarily) at University College London and funded by the Engineering and Physical Sciences Research Council [21]. The tool being developed by i-sense amounts to a ‘third generation point of care diagnostic test’, being a mobile-phone based technology that is designed to enable simultaneous detection of multiple targets (in this case differing strains of influenza) using more accurate biomarkers than older, second generation tests [22]. The technology works by effectively converting the phone’s camera into a spectrometer. A mechanism is fitted over the phone so that light reflected off commercial influenza tests may be collected through the phone’s camera. An app on the phone then analyses data from the camera to establish the diagnosis. To perform the test, the user starts by running a cotton bud around their mouth to gather a good amount of saliva. They then apply that swab to a chemical strip, before inserting the chemical strip into the mechanism attached to the phone, and then using the app on the phone to run the required analysis – after which the app informs the user of the result (e.g. ‘Influenza detected’). The tool analysed in this study was a prototype. At this point, in a full version of the tool, users would be asked whether they would be willing to share the results of their test (without any identifying information) with i-sense researchers and local health providers (for example, the National Health Service). Users could then additionally be asked whether they would also be willing to share geo-location data (e.g. post-code) and perhaps certain other pieces of relevant information (age, sex of the user etc.). Taken together with other data sets, this would allow researchers to map the spread of a disease across a population in real time, thus aiding infectious disease planning. Similar tests are also being developed by i-sense for bacterial infections and HIV/AIDS [23].^1^

As well as generating many of the same ethical considerations that apply to diagnostic tools in general, the self-test kits being developed by i-sense present their own specific set of issues. On the one hand, as a relatively routine and often asymptomatic infection, individuals may be more open to testing for influenza at home, away from professional support. On the other hand, however, influenza can sometimes also be both highly virulent and extremely dangerous, raising questions about how the development of such tools might change our obligations to one another during a pandemic: would self-testing become morally obligatory? The technology’s potential to collect real-time health data also raises important questions around privacy: even where user’s test results are anonymized, when combined with geo-location and other data there is a risk of re-identification following a data breach, especially in areas of low population density. The extent to which researchers’ use of the data can be adequately covered by a process of informed consent is also open to question. Similar to donations to a biobank, it might be information about how the data will be used in the future is not available at the time of donation and hence cannot be disclosed. If so, the research participant would not know the relevant facts of the specific research and would not know to what they are consenting.

To help map this complex and intricate ethical landscape, we adopt an empirical ethics approach [24], combining normative analysis with qualitative research drawn from three focus groups. In this, we bring together two related strands of research: i), a growing bioethical literature on the ethics of diagnostic technologies, and self-testing in particular [14, 17, 25, 26]; and ii), a sociological literature that has sought to understand individuals’ motivation for and experience of self-testing [18, 27, 28].

The structure of the article is as follows. In section two, we further detail our study’s method. In section three we present findings from an initial review of the ethical issues around self-testing, along with findings from three focus groups convened to discuss the ethical desirability of the technologies, their various benefits and drawbacks. In section four we discuss how these findings confirm some of our existing intuitions around the ethics of such technologies, raise new issues both in their favour and against them, and highlight the context-specificity and disease-specificity of our thinking relating to self-testing. Here we also consider how developers might anticipate and attempt to mitigate possible ethical concerns in the way they design such technologies and set out some of the limitations of the study. We conclude in section five.

## Methods

This paper follows an empirical ethics approach to the ethical issues raised by self-test diagnostics. Specifically, we adopt what Molewijk et al. refer to as a ‘theorist’ approach to empirical ethics, wherein data drawn from the research encounter is used primarily as a means to correct or supplement insights derived from normative theorising [29].

Two features characterise our reading of this approach. First, we take moral authority to be located in what is normatively justifiable rather than social practice as such. Hence, we would not consider individuals’ views that a particular practice was ethically acceptable to be definitive proof of its ethical acceptability. Such individuals might be wrong. At the same time, our approach is amenable to making changes to normative judgements or broader normative theory if, say, the empirical data showed certain features of the examined context to have a normative significance previously ignored.

Second, we recognise that qualitative research is not necessarily a path to some general set of facts about individuals’ responses to a given practice, potentially offering only a contingent and partial account of aspects of the participants’ lives [30]. Thus, rather than viewing the empirical parts of this research as establishing some fact of the matter about individuals’ views about the ethical issues around diagnostic technologies, instead our aim is to use such interactions to help us, as ethicists, develop our own understanding of the ethical considerations at play. The thought here, then, is that through what Michael Parker has called ‘encounters with experience’ [31], one is able to develop a more fine-grained appreciation of the ethical landscape than would be possible through abstract philosophical reflection alone.

The method followed in this paper comprises three steps: i) an initial reflection on the potential ethical considerations at play; ii) qualitative research, also designed to elicit perspectives on the ethical problem at hand; and, iii) a revision of the initial reflections in light of an interrogation of the qualitative data. We now describe each of these in greater detail.

### Initial reflection

The first step comprised an initial reflection by two researchers (BR and JW) on the main ethical issues that *might* be raised by self-test diagnostic kits for influenza. This involved, variously: i) an informal review of emerging medical, sociological and ethical literature on both point-of-care and self-test diagnostic technologies published within in the last 20 years, ii) informal discussions with the team designing the i-sense kits, and iii) independent philosophical analysis of the technologies. Where necessary, potential considerations identified in the literature, during conversation, or following independent reflection were subjected to additional research (say, by ‘reading around’ the subject, discussing with colleagues, or applying further philosophical reasoning), before researchers came to a final judgement as to their relevance. Each source of information (literature, discussions, philosophical analysis) guided research into the other two and researchers met regularly to discuss findings. Aiming at inclusivity rather than exclusivity, all considerations taken to be in any way pertinent to the moral acceptability of self-testing kits for influenza were included. These considerations are summarised below in the results section.

### Qualitative research

The qualitative aspect of the research took the form of three focus groups, convened to consider the ethical issues raised by self-testing for influenza, concentrating in particular on the tool being developed by i-sense. Focus groups were chosen over other qualitative research methods (e.g. in-depth interviews) for a few reasons. First, focus groups have been widely used to examine people’s experience of health and social care topics [32–35] and their experience of technology and health [36, 37]. Second, as an ethical enquiry, we were keen to generate opportunities for point-counterpoint discussion and among equals, illuminating the difference in perspectives, where our worry was that in a one-to-one encounter they may have been more likely to defer to the expertise of the researcher, or to feel intimidated by that expertise. Thirdly, the data generated through social interaction of a group are often deeper and richer than those obtained from one to one interviews [38]. Accordingly, focus groups seemed a suitable approach given the relatively broad nature of the enquiry, allowing the group to generate and explore their ideas, in a way which would not be possible in an interview, allowing participants to use their own vocabulary, generate their own ideas and pursue their own priorities, drawing out latent issues [32]. Finally, given many participants were new to the technologies being discussed, it was thought that mini-groups may be preferable to one-to-one encounters as they allowed individual respondents to time and space within the research encounter to reflect on the technology being presented and their ethical opinions – i.e. to think while others were speaking..

In selecting participants for the focus groups, our approach was informed by Ives and Draper’s dictum that in seeking to understand the context of a problem one needs to sensitise oneself ‘to the needs and experiences of those most affected by it’ [39]. As such, the make-up of the three groups was designed first and foremost to reflect individuals’ differing levels of knowledge and familiarity with the technology in question, rather than with respect to any particular demographic group. Further, based on Kruger, focus groups work best when participants share similar characteristics [40]. The three groups comprised:

i) members of the public – which is to say, those entirely new to i-sense technologies but potential future users;
ii) participants involved in the UK FluSurvey project^2^ [41] – those familiar with novel methods of influenza detection, though not necessarily the i-sense technologies;
iii) i-sense administrative staff and researchers – those familiar and knowledgeable about i-sense technologies.

Members of the public were recruited through email advertising, using staff and student mailing lists at University College London and a Healthwatch e-newsletter,^3^ as well as through advertising on Twitter and university-wide posters. FluSurvey participants were recruited from an existing pool of FluSurvey users who have previously voiced an interest in participating in focus groups, known to one of the researchers (CW). i-sense participants were approached through an email advert to all PhD students and staff working on the i-sense project (both from UCL and other universities).

Recruitment took place from October to November 2015. Responses were received from 8 members of the public, 8 FluSurvey users and 10 i-sense researchers. To ensure the perspective offered by each group remained distinctive, researchers checked members of the public had not previously taken part in FluSurvey, or were part of the i-sense project; with FluSurvey users that they had not taken part in i-sense, and with i-sense researchers that they had not taken part in FluSurvey. No such overlaps were found and, due to relatively low numbers, all respondents were recruited. Participants received a £10 gift voucher as an incentive to participate. The study received ethical approval from UCL’s Research Ethics Committee (7543/001).

During the focus groups, researchers presented all participants with a prototype of the i-sense diagnostic technology and explained how it worked (for logistical reasons, it was not possible to do this prior to the group meeting).^4^ Researchers then asked semi-structured, open-ended questions about the possible benefits and concerns of the technology, encouraging participants to talk about their experiences and stimulating them to express their opinion. (A copy of the topic guide can be found in Additional file 1). Where facilitators felt discussions might benefit from a specific question, participants were asked directly about some of the potential benefits and concerns and related ethics that had been identified during the initial review. However, since the purpose of the qualitative stage of the research was to get a fresh perspective on the ethics of technologies, rather than necessarily to ‘test’ the relevancy of the reasons generated by the initial review (that work being done by the researchers themselves, both during the initial review and in the ‘analysis and synthesis’ stage of the research detailed below), facilitators generally avoided leading the discussions in this way wherever possible.

Toward the end of the session, researchers asked participants whether any of their judgements about such technologies would change if a) the technologies were used to diagnose diseases other than influenza; or if b) they were currently in the midst of a pandemic. These questions were based on evidence showing that perception of different diseases, and diseases during pandemic phases can be important for the acceptability of different health interventions [42, 43]. With respect to the latter, participants were asked to imagine themselves in the midst of the 2009 H1N1 pandemic and were shown a video clip of press coverage from the time to remind them of the outbreak.

All three focus groups took place in December 2015. As a result of non-attendance by some respondents, the final groups comprised of 5 members of public, 7 FluSurvey users and 9 i-sense researchers. Each focus group lasted approximately 90 min. Participants were informed about the goal of the study and signed a consent form indicating that they agreed to anonymous extracts being published and to the conversation being audiotaped. Recordings were transcribed verbatim.

### Analysis and synthesis

To safeguard against subjective perception [38], transcripts of the focus groups were analysed independently by each researcher (BR, CW, JW). Researchers undertook a classical context analysis of the qualitative data [44, 45]. This involved a number of stages [46]. Firstly the data were examined, then categorised into risks and benefits. As suggested by Krueger & Casey (2000) ‘analysis begins by going back to the intention of the study’ [47]. As such, the second step was to extract those risks or benefits which had been identified in the initial review. Thirdly, the transcripts were analysed to identify benefits or risks of the technologies which had not been anticipated prior to the focus groups, which were also extracted. Interpretation of data from each focus group, including consideration of the context, frequency, consistency, extensiveness and intensity of discussion were assessed within the research group until consensus was reached. Fourthly, the data were then grouped thematically, based on the questions asked by the researchers which resulted in similar themed comments across the three groups, (see sections 3.1-3.5).

Once researchers had reached a settled view on the data, a new analysis of the ethics of the relevant technologies was undertaken. Part of this involved an assessment of the extent to which participants’ observations expanded the expected range of ethical concerns. In this, researchers followed a broadly ‘coherentist’ approach analogous to one outlined by Ives and Draper [39]. The primary aim of this approach is to reach a type of ‘reflective equilibrium’ [48], specifically one wherein ‘the demands of empirical data and the demands of theory are weighed up against one another’ [39], in order to reach normative conclusions – conclusions that may be local or may have wider application.^5^ We present the outcome of this process in the discussion section.

## Results

### Initial review of potential costs and benefits of self-tests for influenza

Following a review of the medical, sociological and bioethical literature on diagnostic technologies, researchers compiled the following list of potential benefits and concerns around self-tests for influenza (please Table 1 and Table 2 below).

**Table 1:** Potential benefits of self-test diagnostic technologies for influenza

Benefit	Description
Knowledge of one’s health status	Self-test devices enable users to ascertain their health status [17].
Individual health benefit	Diagnostic devises could allow for more effective self-management of health and medical intervention, reducing the risk of disease, slowing or preventing disease development and/or making treatment more effective [17].
Autonomy	Diagnostic devices as a means of allowing persons to take control of the management of their own health [17].
Privacy and anonymity	Self-testing allows for individuals to conduct diagnostic tests anonymously and in private [14, 19].
Improving the doctor-patient relationship	By enabling individuals to take greater control of their health, rather than, say, being dependent on medical practitioners for knowledge of their current health status, self-testing will allow for a better doctor-patient relationship, one wherein both parties are actively involved in decision-making. [20].
Health benefit to others	Assuming individuals change their behaviour once they receive a positive result, rapid diagnosis could help users from unintentionally passing on infection [14].
Improving health awareness	By giving individuals the ability to monitor their own health status, self-testing may lead to a greater awareness of one’s health and of efforts at health promotion. [58].
Facilitation of public health interventions	By enabling more tests to be conducted and test data to be shared with health systems more easily, some diagnostic tests may enable researchers to identify disease ‘hot spots’ and direct public health interventions accordingly.
Social benefit	Laboratory-independence means tests can often be conducted in diverse settings, most notably those with poor health service or laboratory infrastructure.

**Table 2:** Potential concerns with self-test diagnostic technologies for influenza

Concern	Description
Inaccurate diagnoses	Inaccurate diagnostic kits may lead to individuals undertaking harmful behaviours, either as the result of a false positive or a false negative [15, 16].
Inaccurate understanding	Individuals may misunderstand test results or accuracy of those results [17, 18].
Insufficient pre-test support structures	By enabling individuals to diagnose themselves away from health care settings, self-testing kits may mean individuals undertake test without the pre-test counselling by health care professionals and/or counselling services, potentially leading to psychological harm [14, 19, 20].
Insufficient post-test support structures	By enabling individuals to diagnose themselves away from clinical settings, self-testing kits may mean individuals receive test results without the support of health care professionals and/or counselling services [20, 26].
Deter necessary service interactions	By enabling self-diagnosis, self-tests may lead to individuals consulting clinicians about their health less frequently, meaning conditions other than those being tested for are left undiagnosed for longer.
Undermining obligations to others	By performing tests in private, individuals may be less likely to inform others of diagnosis (e.g. contact tracing) [26].
Undermining relationship to others	By supporting an overly individualistic model of health care, self-testing may be inappropriate for health care, which tends to privilege close relationships and human interdependence [17].
Threat of testing becoming mandatory	Danger of self-testing being made obligatory, either legally or through social pressure (e.g. during an epidemic) thereby infringing individuals’ right ‘not to know’ [14, 59].
Privacy breaches through theft of kit	Individuals’ right to privacy may be infringed through theft and/or misuse of the diagnostic kit [26].
Privacy breaches through use of test data	Where test data is stored and analysed remotely, individuals’ right to privacy may be infringed through misuse of data (including onward sale to third parties).
Cost and unequal access	If the cost of the diagnostic technology is sufficiently high, distribution based on ability to pay may exacerbate existing health inequalities [14, 20].

We now present findings from the focus groups on the ethics of self-tests for influenza.

### Potential benefits of self-testing diagnostic technologies for influenza

Upon being presented with the i-sense diagnostic for influenza, participants suggested a range of potential benefits. First, participants from all three focus groups acknowledged that one of the benefits of such tests is that they allowed users to gain first-hand, immediate knowledge of their influenza status. One FluSurvey user, for example, commented ‘It would be really nice to know whether in fact I did have ‘flu or not or whether it was just a man getting excited about a cold’ (F5). However, it was also clear that participants were sceptical about the value of such knowledge in itself. Rather, participants in all three groups argued that such knowledge was only valuable insofar as it made a difference to which treatment options one pursued, or if it led directly to a specific health service intervention. As one member of the public commented:

‘That is actually a good point because you need an action immediately. Instead of just having the diagnosis, like a big ‘yes’ on the screen, you’re positive, it’s like, “Okay, now what?”’ (M2)

Another benefit mentioned both by members of the public and FluSurvey users was that the test could help one avoid unnecessary clinical consultations. Notably, for respondents in both groups this was as much about ensuring that they did not ‘waste’ their doctor’s time, or ‘bother’ them, as it was wasting their own time in consulting a physician unnecessarily.

These more altruistic considerations were also present in respondents’ views about the potential public health benefit of the kits – one feature of the diagnostic tool being developed by i-sense being that it allowed for a ‘real-time’ geographical tracking of influenza ‘hotspots’ as they developed, enabling health service interventions to be directed accordingly. One FluSurvey user noted, for example, that ‘Maybe if you begin to notice there are some outbreaks of flu that it is worth spending the money to campaign to get people who are at risk to make sure they get to the pharmacy or the GP to have a flu jab’ (F1),^6^ others emphasising that this would be particularly beneficial for ‘at risk’ communities. Similarly, one i-sense researcher felt this benefit was significant enough to overcome their normal aversion to sharing data in other contexts:

‘I mean there are other apps that I always am just like no, there's no benefit to [sharing my data] but I can actually appreciate public health benefit.’ (R3)

At the same time, however, it was noticeable that of the three groups, only FluSurvey users noted a more basic benefit of the diagnostic technologies: namely, that they could benefit others by preventing users from unintentionally passing on their infection (see ‘health benefit to others’ consideration in Table 1). The FluSurvey users also noted that, by virtue of the fact they did not require samples to be analysed in lab, such tests were also of particular benefit in countries with poor health services or laboratory infrastructure (‘social benefit’ consideration in Table 1).

‘I know we are talking about it in flu terms but certainly the potential as a diagnostic tool worldwide in somewhere that people who can’t necessarily get to a GP easily or get to a medical resource easily.’ (F2)

None of the participants made any unprompted mention of issues around autonomy, improving the doctor-patient relationship, improving health awareness or the ability to conduct tests in private (neither were they prompted to discuss these issues). However, participants did raise several new benefits researchers previously overlooked. Participants in the FluSurvey group, for example, noticed that the diagnostic technology could help to reassure the ‘worried well’ through a quick ‘all clear’ diagnosis and moreover, such kits might also aid in ‘automation’ of employee medical certificates. i-sense researchers also noted that such diagnostics might prevent the misuse of antibiotics for viral infections. However, it is also worth mentioning that in recognising this benefit, the i-sense researchers also voiced a concern that it might be ‘negative’ for developers to say that one benefit of the technologies is that they prevent people from taking certain treatments when ill (however inappropriate those treatments might be). In turn, this led to a discussion about how developers might build and maintain public trust in self-test kits. Are there some benefits to such technologies that it is better (more prudent) for the developers not to mention?

### Potential concerns of self-testing diagnostic technologies for influenza

As well as these potential benefits of diagnostic technologies for influenza, the focus group participants also expressed a number of possible concerns. One issue for all three groups was the accuracy of the test itself. As well as being concerned about the accuracy of the technology in general, i-sense researchers in particular were concerned that, when used as a self-test, there was an added risk of individuals not using the technology properly, thereby further jeopardizing the validity of the results.

‘As a scientist, I would also consider the accuracy of that test in a home setting because, as you mentioned, some people might not go deep enough [with the swab]. If I see a negative, is it really negative and then I don't do anything.’ (R7)

From this, the i-sense group suggested that the technology should come with a ‘control’, informing the user that the test had been used appropriately (such as with a pregnancy test). In a related discussion, one FluSurvey user suggested that the technology should only ever offer provisional guidance, with a strong recommendation to seek further medical advice:

‘I think the message always has to be whatever the result if you are concerned to talk to your health professional…I think it should never be presented as something which is giving you a cast iron, copper bottom medical advice. It is only a tool that provides guidance for what you might do.’ (F1)

All groups raised the question of how much the technology would cost and/or whether it would be provided free, for example by the National Health Service (NHS). In most cases, this was about whether the technology constituted good value for money for them personally. However, one FluSurvey user also wondered whether free provision of fast diagnostic technologies might also be beneficial to the health provider itself as a matter of technical efficiency:

‘If they aren’t that cheap then if it was in some way tied in or they have an effect such that they do reduce the strain on the NHS then surely the NHS to give them out as a freebie to certain groups.’(F3)

Along with these concerns, one issue that received extensive discussion in all three groups was the risk of privacy breaches through use of test data. In the preamble to the discussion, facilitators explained that users of the diagnostic technologies could choose to share data about their result, themselves and their geographical location with public health researchers and the public health service but that one risk associated with this is that it might threaten individuals’ privacy. Each group responded to this concern differently. Members of the public were relatively relaxed about these risks, arguing ‘for most people they would not care about that much’ (M1) and ‘as long as you keep the name confidential, all the other data can be shared’ (M5). With one exception, both FluSurvey users and i-sense researchers were similarly sanguine about sharing their data with the i-sense group and potentially a public health service more widely for research purposes. However, both groups reacted strongly against the possibility of the data being sold to commercial companies, either as a commodity in itself or for marketing purposes. One FluSurvey user argued:

‘From a personal perspective I think the risk of our personal medical data being sold for commercial purposes you want to talk about risk with something that I am really not comfortable with, I do find that very difficult.’ (F1)

Two further features of this discussion are worth noting. First, far from seeing the sharing of test data with the public health service as a potential threat to privacy, several participants argued that the data ought to be automatically linked with their NHS record, so their physician could be kept abreast of their current influenza status. Second, it was clear that, in the case of some participants, it was not necessarily that they were undaunted about the potential threat to their privacy posed by the i-sense tool as they were resigned to the fact that, at any given moment, their informational privacy was being infringed by various actors and, in that sense, these tests were no different and perhaps better (being for a more worthwhile cause).

None of the participants made any mention of the other issues identified in Table 2, such as the technology undermining of relationship to others, becoming compulsory (either as a matter of legislation or social pressure), or deterring necessary clinical consultations, nor did they make any mention of the risk that one’s privacy might be infringed through theft of kit, the possibility of inaccurate understanding of the test results or the absence of pre- and post- diagnosis counselling, when the kit was used at home.

At the same time, participants from the i-sense group did raise two new concerns around such technologies: first that it might facilitate hypochondria (‘I also think there's a concern, the worried well, people who are not really ill just continually testing, getting increasingly obsessed’ – R4); second that it might change social interactions, in that it may become the norm for individuals to demand another’s health status prior to close contact. Both these considerations appeared to be part of more general concern that in enabling individuals to have quick and easy access to information about their health, self-testing may lead to an ‘unhealthy’ approach to one’s current health status – that it could come to dominate one’s understanding of oneself and others where it should remain in the background. In this way, then, participants identified a possible cost to the technology’s ability to grant true information about one’s health status previously overlooked during the initial review (which had tended to assume that all such knowledge was to the good, the problem being instances in which the diagnostic technology presented false information or when a user misunderstood the information being presented to them).

### Using self-tests to diagnose other diseases and conditions

One of the issues researchers were interested in examining was the extent to which participants’ views about the ethics of self-testing depended on the type of disease being investigated. To investigate this issue, facilitators asked each focus group to consider whether they would change any of their prior responses if the diagnostic tool were for testing diseases other than influenza (examples given included chicken pox, Ebola and HIV/AIDS).

This set of questions prompted a range of responses. First, both members of the public and FluSurvey users argued that one’s moral obligation to prevent passing on infection to others was far stronger with regards to diseases with a high case fatality rate than they were for influenza, in turn, making the technology’s ‘health benefit to others’ a more weighty consideration in its favour. FluSurvey users also judged the fact that self-testing could be employed in countries with poor health care infrastructure (‘social benefit’ consideration in Table 1) to be of greater importance when it was suggested that such tools could be used to diagnose diseases with a greater potential transmission rate or symptom severity than influenza.

Notably, it was also only when it was suggested that similar kits could be used for sexually transmitted diseases such as chlamydia and HIV /AIDS that participants remarked that one benefit of the technology was that it allowed individuals to perform the test in private (‘privacy and anonymity’ consideration in Table 1), something that they had not mentioned with respect to self-testing for influenza. As one FluSurvey user put it:

‘I was thinking along the lines of sexual health, not necessarily HIV but more the chlamydia and gonorrhoea. That might be something that people would prefer to in-house test at home before going to the GP rather than going straight down there.’ (F3)

Changing the disease the diagnostic tested for also changed participants’ views about their associated problems. For example, when asked to imagine that the i-sense tool diagnosed HIV/AIDS, rather than influenza, participants from all three groups were much more concerned about sharing their data with researchers and the attendant risks to their privacy. As one member of the public explained:

‘I think it depends on things that you are worried about. If you have worry about that you are being infected by HIV, then you definitely would not like others to share…your information….[S] omething like a small infection…that’s fine. It depends on different kinds of diseases.’ (M1)

Moreover, it was also only when researchers suggested that the diagnostic technology could be used to diagnose potentially life-threatening diseases like HIV/AIDS that participants began to voice concerns about the lack of pre- and post-diagnosis counselling. As a FluSurvey user put it:

‘I think you would feel very isolated if you got a yes. I think you need more counselling, more background, more help or more something before you take the test. Once you have that and then you get a yes or a no then you can deal with the answer I think.’ (F6)

### Using self-tests during a pandemic

As well as seeking to find out the extent to which participants’ views depended upon the disease being diagnosed, researchers also sought to understand how (if at all) participants’ views about the technology changed in conditions of a pandemic. Faced with this scenario, participants’ intuitions about the benefits and risks of self-testing changed in several ways. First, for one FluSurvey user, the context of a pandemic gave greater significance to the diagnostic information generated by the technology (‘Knowledge of one’s health status’ consideration in Table 1). Participants from all groups also valued the benefits of the technology in limiting the spread of infection much more highly in the case of a pandemic (‘Health benefit to others’ consideration in Table 1). As one member of the public explained:

‘I would do it just to check on me but also to protect the others, because if I have it, then obviously – and it’s not a common cold – then I wouldn’t walk in school…or go to the airport, or do anything of the sort, because I would jeopardise the health of the other people. If I’d do it, I’d do it so that I would stay at home.’ (M4)

Following this altruistic line of thought – with participants again considering how the diagnostic tool might be beneficial for people other than themselves (that is, while imagining themselves as the user) – participants also emphasised the fact that such tests might alleviate pressure on the NHS or health provider by allowing for diagnosis at home (‘Deter unnecessary service interactions’ consideration in Table 1).

In the context of a pandemic, participants from all three groups were also much more open to sharing data about their test results and themselves with public health researchers and the health service in order to help track the spread of the infection; that there was, as one i-sense researcher put it, ‘a sense of solidarity’.

Overall, the response from the group was that in the case of a pandemic, the benefits of such technology became even more valuable and its potential problems were taken to be less concerning. Perhaps the only problem that participants did feel would be exacerbated in pandemic conditions was the potential for the technology to become mandatory or rather, for there to be greater social pressure to use it.

## Discussion

Data gathered during the focus groups simultaneously demands several adjustments to our understanding of the ethics of self-test diagnostic technologies and yields a number of lessons for developers of such technologies. It also brings into focus certain limitations both of this study and, potentially, empirical ethics methodologies in general. In this section, we discuss each of these in turn.

### Changes to our understanding of the ethics of self-test diagnostics demanded by the empirical evidence

Perhaps the most valuable thing to emerge from the focus groups was the addition of five new considerations around the ethics of diagnostic technologies overlooked in our initial review. In drawing our attention to these issues, one can see the value of seeking responses on a given technology from a diverse range of participants and, in particular, participants who approach the technology from a diverse range of perspectives. Although these issues might have been raised by participants from any group, the fact that, for example, the diagnostic tool might prevent the misuse of antibiotics for viral infection was raised by the i-sense researchers seems to point to the value of obtaining views from groups with differing expertise (a reasonable assumption being that the i-sense researchers would normally be better versed in treatment options for influenza and the dangers posed by overuse of antibiotics than a reasonably random selection group of members of the public or FluSurvey users).

One notable feature of the new considerations themselves, perhaps, is that while they all clearly contribute to the ethical acceptability of the technologies being developed by i-sense, none of them seem sufficient to establish the ethical acceptability of such technologies overall. The fact, then, that such technologies might reassure the worried well, does not seem to establish that they are all-things-considered a ‘good thing’; nor the fact that they might alter social interactions for the worse that they are therefore ethically impermissible. This reflects a wider pattern in the group discussion: namely, that none of the participants took any of the considerations for or against the use of the relevant technologies to be decisive in establishing their ethical acceptability. Rather, all such reasons were considered contributory reasons, features whose presence counts towards the overall goodness or badness of some objection or action [49], but which could be traded-off against one another in an overall assessment of the technology’s value. Again, this seems correct.

Another way in which data from the group discussion aids ethical inquiry is insofar as it draws attention to certain issues arising from considerations already flagged in the initial review but which themselves may have previously gone unnoticed. For example, although some participants were perhaps over-zealous in their view that knowledge about one’s health status could only be considered beneficial insofar as it made a difference to one’s future course of action (we can recognise that there might still be an intrinsic value to such knowledge), by making that point they drew attention to the fact that some individuals will only take up such tests where they can see a clear reason to participate. Group discussions were also useful in reminding us that interactions with the health provider come with two sets of opportunity costs: one to the patient and one to the attending physician.

One thing that came through strongly in all three focus groups was just how disease specific participants’ thinking about many of these considerations was, both in the risks and benefits that they picked out as salient and in the weight they accorded to them. Some of these patterns look eminently predictable. We would expect, for example, that the health benefits of diagnostic tests – and, conversely, the need for pre- and post-diagnosis counselling – increase the more severe the symptoms of the disease, or the greater potential transmission rate. However, it also looks like that there is a level of granularity to the ethical acceptability of different technologies that may have been previously neglected. Indeed, in respect to certain considerations, it may be that the ethical desirability of a given diagnostic depends not only on what disease is being tested for but where that test is taking place. Whether a disease carries a social stigma, for example, is likely to vary from society to society, as are perceptions about privacy and concerns over data protection. Data gathered on self-testing in pandemic conditions also reminds us that the patterns of considerations at play can be a matter of ‘when’ the tool is being employed, as much as ‘where’ and ‘about what’.

Findings from the focus groups were also useful in pointing to new opportunities. As reported above, several participants argued that the data from the kit ought to be linked automatically with their NHS record, so their general practitioner could be kept abreast of their current health state. This willingness and indeed expectation that data will flow for the purposes of direct care where appropriate is consistent with broader information governance legislation and policy within the NHS, such as the 2013 Information Governance Review, which concluded that the duty to share information ‘can be as important as the duty to protect patient confidentiality’ [50] and the Health and Social Care (Safety and Quality) Act 2015. The ability for patients to not only see, but also make additions to, their Personal Health Record are scheduled to be available for all NHS patients by 2018, so there is an obvious opportunity to allow self-test data to connect up in this way.^7^

As a final point, it ought to be mentioned that none of the data suggests to us that any of the initial set of benefits and risks detailed in Tables 1 and 2 were either mischaracterised, mislabelled (e.g. as benefits rather than concerns) or irrelevant. Similarly, the fact that participants chose to focus on some considerations rather than others – say, the fact that the test could help one avoid unnecessary clinical consultations over its health benefits in limiting the spread of infection – does not suggest that, as such, we ought to take these as the most pressing issues in considering the various merits and shortcomings of the kits.

### Recommendations for the development of self-test diagnostic technologies for influenza

In furthering our knowledge about the moral considerations at play in evaluating self-test diagnostic technologies, the findings of this research project generate a number of lessons for the future development of app-based influenza diagnostics. The process of coming to a reflective equilibrium about this particular diagnostic tool formed part of a commitment to responsible research and innovation on behalf of the project funding it, and thus we drew on an emerging literature connecting empirical ethics to the responsible research and innovation agenda [51].

Given this focus, the object of our reflective equilibrium was not only the ethical issues raised by influenza diagnostic tools in general, but also the types of ethical and governance challenges that could result from bringing this particular diagnostic tool to market, and how the design of the prototype might be iterated in response to these. One important decision point in the development of this, and any diagnostic tool, is whether the tool would count as a registered medical device, and if so, of what class. If a tool does count as a medical device, it needs to be registered and the test and validation data needs to be available for scrutiny by the regulator. Directive (93/42/EEC) and Directive (98/79/EC), set out the framework within the EU.^8^ The rise of fitness trackers and health apps more generally has created a degree of uncertainty around what counts as a medical device, with a new EU-wide Regulation expected to be adopted soon.^9^

There were more than 165,000 health apps on the market in 2017, of which only a very small percentage were registered as medical devices. Most of these provide general advice and education, or allow self-tracking, but as the New York Attorney General examined in an investigation that led to three app manufacturers being fined, some claim to “measure vital signs and other key health indicators using only a smartphone” without having adequately tested and validated the accuracy of the results [52]. Moreover, earlier work showed that even apps that had undergone a process of appraisal to enter the curated NHS Health Apps Library raised very serious concerns of privacy and data protection [53].

Much of the approach to testing and validation that is required for medical devices for self-testing will also be good practice for diagnostic tool kits that are not medical devices, for instance that they ‘must be designed and manufactured in such a way that they perform appropriately for their intended purpose taking into account the skills and the means available to users and the influence resulting from variation that can reasonably be anticipated in users’ technique and environment’ [54].

We recommend the following, some of which depend on whether or not the tool is to be a medical device:

- First, to minimize the risk of individuals misunderstanding test results, all results should come with a warning as to their accuracy. Given it has been shown people often find it difficult to understand risks when rendered in percentages [55], these ought to be presented in natural frequencies. (For example, ‘For two in every 100 people, this result will be wrong’; as opposed to ‘This test is 98% accurate’). It may also be prudent for such information to differentiate between the sensitivity and specificity, which is to say, between (a) how likely is it that given a positive result you actually have flu, and (b) how likely is it that given a negative result, you do not have the flu. At the design stage, empirical work should be undertaken to determine the tolerance for false positives and false negatives within the target population for each disease to be tested. Given the contextual nature of individuals’ responses in our focus groups, these tolerances are likely to be context and disease specific.
- Second, in order to ensure tests are of maximum utility, designers should consider ways in which the app might alert users to recommended courses of action, rather than simply presenting users with a diagnostic result. For example, the app could give users information about how they might avoid infecting others and remind them of their moral obligations in this regard. However, it is important to be clear about the nature of any advice given, and whether such advice provision would require the tool to be a registered medical device.
- Third, where it is technically feasible for data to flow securely, patients should be given the option for the data from the self-test to be added to their personal health record. Any choice to share the information to the individual’s personal health record should be entirely separate from consent to share the information more widely.
- Fourth, while running the app, users should be encouraged to share their data for the purposes of epidemiological research and disease surveillance. Strict protocols should also be put in place to ensure that consent for diagnosis and direct care is kept separate from consent for research and disease surveillance. Users should be able to withhold their consent to sharing their data at any stage in the process (and be given some choices about what information to share), even if this results in a less useful data set. Consent for the use of such data for research purposes should not be mistaken for consent for such data to be sold to private companies, either as a commodity or for marketing purposes.
- Fifth, to guard against misconceptions about the technology and to build public trust, designers need to be clear and transparent about how the technology works, who the data is held by, whom any shared data is shared with and what purposes such data is put to. Following participants’ discussion about whether there are some benefits it is better for developers not to advertise (Section 3.2), we believe developers should also be open and transparent about all the potential benefits and drawbacks of such technologies, even when that challenges deep-seated behaviours among the public. If, then, a diagnostic kit for influenza might help to slow the rise of antibiotic resistance by dissuading individuals from taking antibiotics unnecessarily, developers should highlight that fact. .

### Limitations of the study

We take the findings presented above to showcase one example in which qualitative research methods can be used to supplement and augment traditional philosophical reflection. At the same time, however, the study has certain limitations.

First, although recruiting participants from i-sense staff and students allowed researchers to gather data from those with intimate knowledge of the relevant technologies, it also incurred certain costs. Three of the nine i-sense participants were known to the facilitators and all could be said to have at least some form of professional relationship with one another, simply by virtue of belonging to the same research group. Despite facilitators efforts to encourage participants to ‘speak freely’ in all focus groups, these facts may have had some bearing on the opinions offered (for example, it may be that staff felt they could not be as critical of the technology as they would, say, in a private conversation).

The quality of the discussion within each of the focus groups was also not always as high as it might have been. On the one hand, as detailed at length above, all three focus groups generated extremely useful information, giving voice to a number of benefits and concerns which had not been picked up in the initial review, as well as providing other data that has been vital in designing recommendations about how we ought to govern self-test diagnostics in the future. In this sense, the value of the qualitative aspects of the research – and the project of a more ‘empirical’ ethics more widely – is entirely vindicated. However, at the same time, participants often failed to engage a rigorous ethical assessment of the technologies at hand. For example, at times, participants offered views about the benefits and risks of the diagnostic that depended on false or questionable or assumptions about treatment options (such as that it would be useful because, once one received a positive result, one would be able to seek an antibiotic prescription, or a flu jab). At other times, respondents appeared to engage in fallacious reasoning. For example, at one point participants argued that even if those handling data generated by the diagnostic kit violated their right to privacy – say, by sharing it with third-parties without their consent – such violations were not of any normative significance because their right to privacy was already being regularly violated by numerous other parties; whereas the mere fact that one’s right to privacy is already being violated by others would not make it any less ethically unacceptable for i-sense, or any other designer of diagnostic technologies, to violate one’s right to privacy.^10^

To a certain extent, this might be seen as an inevitable consequence of a flaw in the study’s design. As set out in the Methods section, during the focus groups, facilitators deliberately refrained from intervening in the participants’ discussion wherever possible. This was for a couple of reasons: first, as intimated above, since we were keen to get a fresh perspective on the ethics of technologies, facilitators were reluctant to ask any leading questions. More broadly, however, facilitators also wanted to cultivate an atmosphere in which participants felt they could speak freely; the thought being to encourage all ideas as ‘good ideas’ in the first instance and then weed out the good from the bad when the data was analysed (as we have done).

Of course, it might be argued that the research encounter would have been more productive, and produced a richer, more rigorous discussion of the issues at hand, if facilitators had adopted a more Socratic approach to the focus groups, perhaps challenging participants on the merits of their ideas, or pointing out when they were engaging in fallacious reasoning. Such an approach has a rich pedigree in empirical ethics, having been pioneered by Alderson, Farsides and Williams in the early 2000s [56], and endorsed more recently by Dunn, Sheehan, Hope and Parker [57]. However, in this particular case, we maintain that such an approach would have been more likely to close down discussions rather than open them up. It ought to be remembered that participants from two out of the three focus groups in our project had no prior knowledge of the relevant topic whatsoever (the technology being a prototype); were not acknowledged experts in the field (who, as such, might feel confident about offering their opinion even among strangers); were completely unknown to each other prior to the meeting - and thus were in the process of establishing their social status within the group during the discussion itself; and, perhaps most importantly, were considering several of the questions before them for the very first time. All this, in our view, made it highly unlikely that participants would respond well to having whatever nascent opinions they offered publicly challenged in an unusual social scenario by what would have been, for them, the only ‘experts’ in the room.

Overall, we believe these findings point to a deep dilemma for the project of empirical ethics. As Ives and Draper argue, we want to sensitize our ethics to the needs and experiences of the individuals most affected by the kinds of matters we are investigating. Yet here we face a problem. On the one hand, those who are most affected by such issues are not always those who are best trained in articulating their ethical import. On the other hand, those who are best trained in articulating the ethical issues at stake – which is to say, ethicists – are rarely those who are most affected. What we require, then, is a way of constructing a research encounter in such a way that it *either* enables ‘those who are most affected’ to engage in a rigorous assessment of the ethical issues, or enables ‘those who are best trained’ to gain a deeper knowledge of what it is like to be affected by the relevant question.

## Conclusion

There seems good reason to think that diagnosis through a self-test kit, such as that being developed by i-sense for influenza, may 1-day become the norm for a range of diseases and conditions. Yet the ethical issues around such technologies remains unclear. In this article we have sought to provide a near-comprehensive map of the ethical issues around one set of technologies in particular: namely, third-generation, self-testing kits for influenza. In so doing, we have adopted a relatively new method in bioethics, combining traditional philosophical analysis with findings from qualitative research. Such sociological research has been invaluable in identifying new considerations at play in our ethical thinking about diagnostic technologies, confirming and augmenting our understanding of issues already identified, and stimulating further ethical reflection. However, our study has also revealed, perhaps, certain difficulties inherent in the empirical ethics project – at least as it applies to the use of focus groups. Such limitations do not undermine our findings but they do show the difficulties involved in constructing research encounters that are able to both elicit the views and experiences of those engaged in ethical dilemmas, while simultaneously satisfying the standards of a philosophical discussion.

## Additional file

Additional file 1: Building and Maintaining Public Trust in Early Warning Sensing Systems for Influenza - Topic Guide. (DOCX 20 kb)

## Footnotes

1 Further information on the diagnostic tool and updates on i-sense’s progress are available at http://www.i-sense.org.uk.

2 FluSurvey is an online service that any member of the UK public can log onto to report flu like symptoms they may experience during the winter months. The data is used by researchers at Public Health England and London School of Hygiene and Tropical Medicine to monitor flu trends in the UK. For more information see http://www.flusurvey.org.uk.

3 Healthwatch England is the national consumer champion in health and care, with statutory powers to ensure the voice of the consumer is strengthened and heard by those who commission, deliver and regulate health and care services. For more information see http://www.healthwatch.co.uk.

4 To keep baseline conditions the same across all three focus groups, facilitators also gave the same demonstration to participants from the i-sense group, although by virtue of their backgrounds, most of them were already familiar with the diagnostic kit and its workings.

5 Dunn et al. have raised concerns about combining an empirical ethics with a ‘coherentist’ approach to moral philosophy (Dunn M, Sheehan M, Hope T, Parker M. Toward methodological innovation in empirical ethics research. *Cambridge Quarterly of Healthcare Ethics*. 2012; 21(4): 466–80); one concern being it fails to recognise the qualitative research as a journey towards particularity rather than universality (Carter SM. Beware dichotomies and grand abstractions: Attending to particularity and practice in empirical bioethics. *American Journal of Bioethics* 2009;9:76–7). However, to respond briefly, it is important to differentiate here between coherentist approaches to the *analysis* of empirical data and coherentist approaches to the wider *metaethical significance* of such data. In adopting a method of reflective equilibrium, the thought is *not* that any such data will allow us to understand the way in which principles and intuitions coalesce in general. Instead, it is that such a method allows us a way of reaching a perspective on the normative meaning of that datum. Thus, while we can agree with Dunn et al. that such data is unlikely to ‘set us on a path to coherence’ with regards to how principles and intuitions coalesce in general, *that* does not mean a method of reflective equilibrium in evaluating the normative meaning of the data in question.

6 It would probably be too late for a flu jab at this stage which need to be done in advance of contracting the strain. We discuss the implications of participants’ false beliefs below in Section 4.3.

7 While it is easy to see the potential benefits of this kind of link, it also raises a number of questions about how it might alter the doctor-patient relationship that are ripe for further exploration: the relationship might change from one in which the patient seeks medical advice in discrete consultations (much as one might go to a mechanic upon finding a fault with one’s car); to a relationship of perpetual surveillance, in which the doctor (or health service) is kept informed one’s health status in real-time.

8 This framework is broadly similar to that in the US, but it should not be presupposed that if something is a medical device in one regulatory framework it would count as a medical device in another.

9 Perhaps one might argue that, as far as violations go, the potential violation incurred through misuse of one’s diagnostic data would be a relatively minor one but this did not seem to be the participants’ thought. Rather, their thought seemed to be that since such violations had become the norm they therefore no longer presented an ethical wrong.

10 For progress reports, see: European Commission Website. Revisions of Medical Device Directives. Available at: http://ec.europa.eu/growth/sectors/medical-devices/regulatory-framework/revision_en. (Accessed 30 March 2017)

## References

[1] Phillips S, Wyndham L, Shaw J, Walker SF (1988). How accurately does the Reflotron dry-chemistry system measure plasma total cholesterol levels when used as a community-screening device. Med J Aust.

[2] Elusiyan JB, Adeodu OO, Adejuyigbe EA (2006). Evaluating the validity of a bedside method of detecting hypoglycemia in children. Pediatr Emerg Care.

[3] Pollock N, Rolland JP, Kumar S (2012). A paper-based multiplexed transaminase test for low-cost, point-of-care liver function testing. Sci Trans Med.

[4] Kassler WJ, Haley C, Jones WK, Gerber AR, Kennedy EJ, George JR (1995). Performance of a rapid, on-site human immunodeficiency virus antibody assay in a public health setting. J Clin Microbiol.

[5] Hislop J, Quayyum Z, Flett G, Boachie C, Fraser C, Mowatt G (2010). Systematic review of the clinical effectiveness and cost-effectiveness of rapid point-of-care tests for the detection of genital chlamydia infection in women and men. Health Technol Assess.

[6] Jarvis JN, Percival A, Bauman S (2011). Evaluation of a novel point-of-care cryptococcal antigen test on serum, plasma, and urine from patients with HIV-associated cryptococcal meningitis. Clin Infect Dis.

[7] Mills LA, Kagaayi J, Makigozi G (2010). Utility of a point-of-care malaria rapid diagnostic test for excluding malaria as the cause of fever among HIV-positive adults in rural Rakai, Uganda. Am J Trop Med Hyg.

[8] Shivkumar S, Peeling R, Jafari Y, Joseph L, Pai NP (2012). Accuracy of rapid and point-of-care screening tests for hepatitis C: a systematic review and meta-analysis. Ann Intern Med.

[9] Boehme CC, Nabeta P, Hillemann D (2010). Rapid molecular detection of tuberculosis and rifampin resistance. New Engl J Med.

[10] Lawn SD, Kerkoff AD, Vogt M, Wood M (2012). Diagnostic accuracy of a low-cost, urine antigen, point-of-care screening assay for HIV-associated pulmonary tuberculosis before antiretroviral therapy: a descriptive study. Lancet Infect Dis.

[11] Ryan A, Wilson S, Greenfield S (2006). Range of self-tests available to buy in the United Kingdom: an Internet survey. J Public Health.

[12] Drain PK, Hyle EP, Noubary F (2014). Diagnostic point-of-care tests in resource-limited settings. Lancet Infect Dis.

[13] Pai NP, Vadnais C, Denkinger C, Engel N, Pai M (2012). Point-of-Care Testing for Infectious Diseases: Diversity, Complexity, and Barriers in Low- And Middle-Income Countries. Plos Med.

[14] Youngs J, Hooper C (2015). Ethical implications of HIV self-testing. J Med Ethics.

[15] Haddow L, Robinson A (2005). A Case of a false positive result on a home HIV test kit obtained on the internet [letter]. Sex Transm Infect.

[16] Ryan A, Greenfield S, McManus R, Wilson S (2006). Selfcare – has DIY gone too far?. Br J Gen Prac.

[17] Kearns AJ, O’Mathúna DP, Scott PA (2010). Diagnostic self-testing: autonomous choices and relational responsibilities. Bioethics.

[18] Ickenroth MHP, Grispen JEJ, Ronda G (2011). Motivation and experiences of self-testers regarding tests for cardiovascular risk factors. Health Expect.

[19] Krause J, Subklew-Sehume F, Kenyon C (2013). Acceptability of HIV self-testing: a systematic literature review. BMC Public Health.

[20] Modra L (2006). Prenatal genetic testing kits sold at your local pharmacy: promoting autonomy or promoting confusion?. Bioethics.

[21] i-sense Website. https://www.i-sense.org.uk/ (Accessed 19 Jan 2016).

[22] Jani IV, Peter TF (2013). How point-of-care testing could drive innovation in global health. N Engl J Med.

[23] i-sense Annual Report 2014–2015. Available at: https://www.i-sense.org.uk/news/i-sense-annual-report-20142015 (Accessed 20 Jan 2016)

[24] Richardson JR. Empirical ethics versus analytical orthodoxy: two contrasting bases for the reallocation of resources. Centre for Health Program Evaluation; 2000.

[25] Frith L (2005). HIV testing and informed consent. J Med Ethics.

[26] Mcgee S (2011). Mobile contact tracing and counseling for STI’s: there’s not an app for that. Am J Bioethi.

[27] Grispen JEJ, Ronda G, Dinant G-J (2011). To test or not to test: a cross-sectional survey of the psychosocial determinants of self-testing for cholesterol, glucose, and HIV. BMC Public Health.

[28] Phillips KA, Chen JL (2003). Willingness to use instant home HIV tests: data from the California Behavioral Risk Factor Surveillance Survey. Am J Prev Med.

[29] Molewijk B, Stiggelbout AM, Otten W, Dupuis HM, Kievit J (2004). Empirical data and moral theory. A plea for integrated empirical ethics. Med Health Care Phil.

[30] Dunn M, Ives J (2009). Methodology, epistemology, and empirical bioethics research: a constructive/ist commentary. Am J Bioethics.

[31] Michael Parker quoted in Ives J, ‘Encounters with experience’: empirical bioethics and the future. Health Care Anal. 2008;16(1):1–6. 10.1007/s10728-007-0077-1 18080836

[32] Kitzinger J (1995). Qualitative research. Introducing focus groups. BMJ.

[33] Wilkinson S (1998). Focus groups in health research: exploring the meanings of health and illness. J Health Psychol.

[34] Richardson CA, Rabiee F (2001). A question of access: an exploration of the factors that influence the health of young males aged 15 to 19 living in Corby and their use of health care services. Health Educ J.

[35] van Dillen SM, Hiddink GJ, Koelen MA, de Graaf C, van Woerkum CM (2003). Understanding nutrition communication between health professionals and consumers: development of a model for nutrition awareness based on qualitative consumer research. Am J Clin Nutr.

[36] Skinner H, Biscope S, Poland B, Goldberg E (2003). How adolescents use technology for health information: implications for health professionals from focus group studies. J Med Internet Res.

[37] Eysenbach G, Köhler C (2002). How do consumers search for and appraise health information on the world wide web? Qualitative study using focus groups, usability tests, and in-depth interviews. BMJ.

[38] Rabiee F (2004). Focus-group interview and data analysis. Proc Nutr Soc.

[39] Ives J, Draper H (2009). Appropriate methodologies for empirical bioethics: it's all relative. Bioethics.

[40] Krueger R (1994). Focus groups. A practical guide for applied research.

[41] FluSurvey Website. https://flusurvey.org.uk/ (Accessed 22 Jan 2016).

[42] Tracy CS, Rea E, Upshur RE (2009). Public perceptions of quarantine: community-based telephone survey following an infectious disease outbreak. BMC Public Health.

[43] de Zwart O, Veldhuijzen IK, Elam G, Aro AR, Abraham T, Bishop GD, Brug J (2009). Perceived threat, risk perception, and efficacy beliefs related to SARS and other (emerging) infectious diseases: results of an international survey. Int J Behav Med.

[44] Holsti OR (1969). Content analysis. Handb Soc Psychol.

[45] Weber RP (1990). Basic content analysis.

[46] Yin RK (1989). Case study research: Design and methods, Revised ed.

[47] Krueger RC, Casey MA (2000). Focus Groups: a practical guide for applied research.

[48] Rawls J (1971). A Theory of Justice.

[49] Dancy J (2004). Ethics Without Principles.

[50] Caldicott F. 2013. Information: To share or not to share? The Information Governance Review. Available at https://www.gov.uk/government/publications/the-information-governance-review. Accessed 21 Mar 2016.

[51] Gardner J, Williams C (2015). Responsible research and innovation: a manifesto for empirical ethics?. Clin Ethics.

[52] New York State Office of the Attorney General Website. A.G. Schneiderman announces settlements with three mobile health application developers for misleading marketing and privacy practices. Available at: https://ag.ny.gov/press-release/ag-schneiderman-announces-settlements-three-mobile-health-application-developers (Accessed 30 Mar 2017).

[53] Huckvale K, Prieto JT, Tilney M, Benghozi PJ, Car J (2015). Unaddressed privacy risks in accredited health and wellness apps: a cross-sectional systematic assessment. BMC Med.

[54] Directive 98/79/EC of the European Parliament and of the Council of 27 October 1998 on in vitro diagnostic medical devices. Annexe 1 B.7 ‘Requirements for devices for self-testing’.

[55] Gigerenzer G, Edwards A (2003). Simple tools for understanding risks: from innumeracy to insight. Br Med J.

[56] Alderson P, Farsides B, Williams C (2002). Examining ethics in practice: health service professionals’ evaluations of in-hospital ethics seminars. Nurs Ethics.

[57] Dunn M, Sheehan M, Hope T, Parker M (2012). Toward methodological innovation in empirical ethics research. Camb Q Healthc Ethics.

[58] Cappelen AW, Norheim OF (2005). Responsibility in health care: a liberal egalitarian approach. J Med Ethics.

[59] Chadwick R, Levitt M, Shickle D (2014). The right to know and the right not to know: genetic privacy and responsibility.

