# Supplementary material for: Self-tests for influenza: an empirical ethics investigation
Source: BMC Med Ethics. 2017 May 5;18:33. doi: 10.1186/s12910-017-0192-y (PMC5420160; doi:10.1186/s12910-017-0192-y)
Supplement: Additional file 1: — Building and Maintaining Public Trust in Early Warning Sensing Systems for Influenza - Topic Guide. (DOCX 20 kb) [file 12910_2017_192_MOESM1_ESM.docx]

Building and Maintaining Public Trust in Early Warning Sensing Systems for Influenza

Topic Guide

Welcome (15 minutes)

*Brief reminder of the aims and objectives of the i-sense project and the ethics sub-project (Participants will already have been sent an information sheet about this in advance).*

*Aims of the qualitative research/focus groups*

*Handing out of the information sheets and consent forms*

Part One: The Diagnostic Kits and Influenza (40 minutes)

*Brief ‘show and tell’ of the diagnostic kits being developed by the i-sense team, with a short description of how the technologies work, including stages of consent.*

**Questions** (*Probes if time*):

1. If the kind of kits being developed by the I-sense were available at your local pharmacy, would you buy them?
2. Would you feel more comfortable if a health professional did the test rather than you? If so, why?
3. Would you have any concerns about sharing data about your test with i-sense by uploading your test results on the mobile app?
   1. *Probe – would you also be happy to upload information about your location (e.g. the first part of your postcode)?*
   2. *Probe – what about more specific data (e.g. date of birth, male/female, phone number, whole postcode, house number etc)?*
4. Would you have any concerns with the i-sense team sharing data from the app with other researchers/public health physicians within the NHS? If so, why?

Part Two: The Diagnostic Kits and Other Diseases (15 minutes)

1. At present, i-sense are trying to develop diagnostic kits for influenza. However, if i-sense were able to develop kits for diagnosing other kinds of diseases, would you be interesting in using these? Are there any diseases you wouldn’t want to use an i-sense kit for?
   1. *If necessary give examples – e.g. Childhood diseases (mumps); Sexually transmitted diseases (herpes, gonorrhoea); Low impact diseases (common cold); High impact diseases (ebola); Easily manifesting/identified diseases (measles, chickenpox)*

Part Three: The Diagnostic Kits in a Pandemic (15 minutes)

Sometimes the way we think about issues like privacy and the sharing of data can be influenced by the context. We would like you now to imagine the following scenario:

*Play video on Swine Flu.*

1. Imagining you were in the midst of this kind of pandemic, would any of your attitudes around home-testing, or sharing your data change? If so how?
   1. *Probe on stigma, quarantine etc*.
   2. *Would you want to check your children?*
      1. *Syracuse principle.*

General Discussion (5 minutes)

1. What concerns do you have about sharing health related data online?
2. What could be done to improve your confidence in these technologies?
3. In general, do you share other pieces of data on line / through apps? Do you think that health data is more confidential? Do you have more concerns about sharing this sort of data than any other pieces of info?
4. Is there anything else that concerns you about the i-sense research?
